# Prevalence of Bacteria of Genus Actinomyces in Persistent Extraradicular Lesions—Systematic Review

**DOI:** 10.3390/jcm9020457

**Published:** 2020-02-07

**Authors:** Mario Dioguardi, Vito Crincoli, Luigi Laino, Mario Alovisi, Diego Sovereto, Lorenzo Lo Muzio, Giuseppe Troiano

**Affiliations:** 1 Department of Clinical and Experimental Medicine, University of Foggia, Via Rovelli 50, 71122 Foggia, Italy; diego_sovereto.546709@unifg.it (D.S.); lorenzo.lomuzio@unifg.it (L.L.M.); giuseppe.troiano@unifg.it (G.T.); 2 Department of Basic Medical Sciences, Neurosciences and Sensory Organs, Division of Complex Operating Unit of Dentistry, “Aldo Moro” University of Bari, Piazza G. Cesare 11, 70124 Bari, Italy; vito.crincoli@uniba.it; 3 Multidisciplinary Department of Medical-Surgical and Odontostomatological Specialties, University of Campania “Luigi Vanvitelli”, 80121 Naples, Italy; luigi.laino@unicampania.it; 4 Department of Surgical Sciences, Dental School, University of Turin, 10126 Torino, Italy; mario.alovisi@unito.it

**Keywords:** endodontic, infections, root canal therapy, actinomyces

## Abstract

*Actinomyces* are anaerobic, rod-shaped, Gram-positive bacteria. They are associated with persistent extraradicular endodontic infections, with possible involvement of the soft tissues of the maxillofacial district. Many studies reported conflicting data on the presence of bacteria of the genus *Actinomyces* in endodontic infections. The aim of this systematic review of the literature was to determine the real prevalence of such bacteria in primary and/or secondary endodontic infections and in cases of persistence with extraradicular involvement. This systematic review was performed according to the PRISMA protocol. A search was carried out through the Scopus and PubMed databases of potentially eligible articles through the use of appropriate keywords. The literature research resulted in preliminary 2240 records which, after the elimination of overlaps and the application of inclusion and exclusion criteria, led to the inclusion of 46 articles focusing on three outcomes (primary outcome: number of teeth with the presence of a persistent extraradicular infection in which the presence of *Actinomyces* was ascertained; secondary outcome: number of teeth with endodontic infection in which the presence of *Actinomyces* was assessed; tertiary outcome: difference in the prevalence of bacteria of the genus *Actinomyces* between primary endodontic infections and secondary endodontic infections). Results of the meta-analysis show how bacteria of the genus *Actinomyces* are present in primary and secondary intraradicular infections and in those with persistence with a prevalence (ratio between teeth with *actinomyces* and teeth with infection) ranging from 0.091 up to 0.130 depending on the subgroups analyzed.

## 1. Introduction

Endodontic lesions may represent a consequence of the invasion of the endodontic space by bacteria. Such micro-organisms may enter the canalicular spaces through carious lesions, traumatic lesions, and periodontal lesions (endo-perio lesions) and determine pathologies such as serous and purulent pulpitis, dental necrosis, and acute and chronic apical periodontitis.

Primary endodontic infection of a tooth can be resolved through endodontic treatment with canal disinfection and sealing of the endodontic system using thermoplastic materials such as gutta-percha [1] and with the aid of epoxy resins or zinc oxide-based eugenol cements [2].

Sometimes due to either incomplete cleaning and disinfection of the canals and the lack of an apical seal, the endodontic treatment can fail in its purposes, and the residual infection can lead to a persistent apical infection [3].

The bacteria *Enterococcus faecalis* is considered the main cause for intraradicular apical persistence infections and endodontic failures; nevertheless, often an endodontic retreatment can determine the remission of the disease [4].

Intraradicular infections sustained by *Enterococci* may be sustained by an important component of extraradicular infection [5]. This last one may be: dependent on an intraradicular infection (generally following endodontic retreatment), i.e., with the remission of the intraradicular infection, the extraradicular infection is eradicated; or independent, when the infection persists despite endodontic treatment, and the apical outer surface of the roots is covered with bacterial biofilms sometimes in filamentous aggregates. Bacteria such as *Actinomyces* and *Propionibacterium* are among those responsible for persistent extraradicular infections [6].

Extradicular infections represent one of the potential causes leading the loss of the dental elements following the failure of both endodontic treatment and retreatment. The abscess can also involve the submandibular and sublinguals lodges, as well as the maxillary sinuses, and can create cutaneous fistulous tracts [7].

Several studies identified bacteria of the genus *Actinomyces* and *Propionibacteium* in extraradicular infections. Ricucci et al. reported in different reports [8,9,10,11] seven cases of persistent infection in radiographically correctly endodontically treated teeth. The histological examination detected the presence of filamentous bacteria (compatible with histological diagnosis of actinomycosis), involving the extraradicular surface of the teeth in continuity with the intraradicular infection, also highlighting the presence of bacterial biofilm that from the external surface involves the endodontic space through the involvement of the lateral canals that can independently sustain the infection of the root.

Focusing on endodontic infections, Claesson et al. 2017 showed the presence of *Actnomyces* (*A. radiscents*) in 16 out of 926 radicular apexes, out of a total of 601 patients in 7 years. In addition, five of the 16 patients with *Actinomyces* had abscesses with fistula persistence, and the same authors reported the first case of persistent apical lesion from *A. haliotis* [12].

Sousa identified 20 extraradicular persistent lesions in 633 cases after endodontic treatment and retreatment; those 20 cases underwent apical surgery, and the subsequent SEM analysis revealed the presence of bacterial biofilm compatible with extraradicular infections without adding information on the presence of *Actinomyces* [13].

In 2012, Wang showed the presence of *Actinomyces* in 11 out of 13 apices, against eight involvements of *Propionibacterium* and five instances of *Streptococcus* [14].

This research provides data on the prevalence of *Actinomyces* in slightly different persistent endodontic lesions.

To our knowledge, no other systematic reviews have been, up today, conducted with cumulative meta-analysis on the presence of *Actinomyces* in persistent extraradicular infections. A previous review focusing on the prevalence of bacteria in endodontic failures identified *Enterococcus faecalis* among the main culprits of failures, highlighting the main bacteria that support endodontic primary infections [15].

The present review aims to provide data on the prevalence of bacteria of the *Actinomyces* genus, on persistent extraradicular lesions (primary outcome) and on endodontic lesion (secondary outcome), giving more information on endodontic infections that involve the external radicular surface (formation of filamentous bacterial aggregates). Moreover, an exact knowledge of the prevalence of cases of extraradicular infections independent of *Actinomyces*-supported intraradicular infection will alert the dentist to the possibility that endodontic retreatment supported by antibiotic therapy is ineffective in resolving the pathology that instead requires surgical extraction therapy or apicectomy.

## 2. Materials and Methods

The following systematic review was conducted based on the indications of the Preferred Reporting Items for Systematic Reviews and Meta-Analyses (PRISMA) statement [16]. After an initial screening phase performed on abstracts identified on the evaluated databases, the potentially eligible articles are qualitatively evaluated in order to investigate the role of bacteria of genus *Actinomyces* in endodontic infections and persistent extraradicular infections on endodontically treated teeth.

### 2.1. Eligibility Criteria and Research Methodology

The studies taken into consideration were in vitro and clinical studies, concerning the subject of infections and persistent endodontic lesions on teeth that already have an endodontic treatment object in particular. Articles dealing with the role of *Actinomyces* in the infection of the external root surface conducted in recent years and published in English were considered potentially eligible. In addition, bibliographies of previously published systematic reviews on similar topics were checked in order to find articles for potential inclusion in this study.

It was decided to focus on articles published in the last 40 years, since the techniques of disinfection, shaping, and sealing in endodontic treatments have radically changed, and data on the prevalence of studies prior to 1979 would already represent a bias for inclusion in the meta-analysis. Moreover, the identification systems of bacteria and of micro-organisms have recently improved, and new bacterial spaces are always identified.

Articles considered to be potentially eligible are those studies that talk about of the role of bacteria in endodontic infections with particular attention to selecting articles dealing with the role of *Actinomyces* in persistent extraradicular infections.

The potentially eligible articles were finally subjected to a full-text analysis to verify their eligibility for inclusion in both qualitative and quantitative analysis.

The inclusion and exclusion criteria applied in the full-text analysis are the following:

- Include all those studies that have identified *Actinomyces* on the external radiculatum surface of the dental roots in teeth with persistent lesions previously treated by means of endodontic therapy;
- Include all those articles that have identified bacteria in persistent endodontic lesions after retreatment with extraradicular involvement;
- Include all articles that have analyzed the presence of *Actinomyces* infections in endodontic lesions (secondary outcome);
- The exclusion criteria are to exclude all those studies and articles that deal only with case reports and reviews;
- Include articles performed on a population larger than twenty teeth;
- Exclude all those studies that did not search for the presence of *Actinomyces* in the endodontic setting and that do not report data on the prevalence or incidence of *Actinomyces*.

Studies have been identified through bibliographic research on electronic databases. The literature search was conducted on the search engines “PubMed” and “Scopus”. The search on the providers was conducted between 1 November 2019 and 10 September 2019 and the last search for a partial update of the literature was conducted on 15 November 2019. Details about search terms and combination strategies used for the literature research are reported in Table 1.

### 2.2. Screening Methodology

The obtained search records were subsequently examined by two independent reviewers (M.D. and D.S.), and a third reviewer (G.T.) acted as a decision maker in case of disagreement between the two reviewers. The screening included the analysis of the title and the abstract to eliminate the records not related to the topics of the review. After the screening phase, the overlaps were removed and the complete texts of the articles were analyzed, from which the ones eligible for the qualitative analysis and the inclusion in the meta-analysis for the two outcomes were identified. Data sought by the two reviewers in the included studies were:

(1) Primary outcome: number of teeth with the presence of a persistent extraradicular infection in which the presence of *Actinomyces* has been ascertained;
(2) Secondary outcome: number of teeth with endodontic infection in which the presence of *Actinomyces* has been ascertained;
(3) Tertiary outcome difference in the prevalence of bacteria of the genus *Actinomyces* between primary endodontic infections and secondary endodontic infections.

### 2.3. Risk of Bias Assessment and Planned Methods for Analysis

The Newcastle–Ottawa scale for case-control study was used to assess the risk of bias in the included studies. Meta-analysis for the primary and secondary outcomes was performed by random effects model with the DerSimonian–Liard method calculating the pooled proportion (PP) of the prevalence of endodontic infections using the software Open Meta-Analyst version 10 (Tufts University, Medford, MA, USA). Moreover, quantitative analysis for the tertiary outcome was performed with the software Reviewer Manager 5.3 (Cochrane collaboration, Copenhagen, Denmark) [17]. In particular, pooled odds ratios (OR) and its 95% confidence intervals were calculated, and the inverse of variance test was applied to test for differences in overall effects between groups. The presence of heterogeneity was assessed by calculating the Higgins Index (*I*^2^); if such measure proved to be higher than 50%, the rate of heterogeneity was considered high. Pooled results of the meta-analysis were represented by forest plots for each of the analyzed outcomes.

## 3. Results

A total of 2240 records were identified on Pubmed and Scopus. After the initial screening phase, the elimination of overlaps and application of the inclusion and exclusion criteria, the following articles were obtained for the three outcomes:

- six articles for the primary outcome: Persoon et al. 2017 [18], Esteves et al. 2017 [19], Sunde et al. 2002 [20], Hirshberg et al. 2003 [21], Zhang et al. 2010 [22], Signoretti et al. 2013 [23];
- 46 articles for the secondary outcome: Pourhajibagher et al. 2018 [24], Lysakowska et al. 2016 [25], Halbauer et al. 2013 [26], Signoretti et al. 2013 [23], Niazi et al. 2010 [27], Fujii et al. 2009 [28], Vianna et al. 2007 [29], Chavez de Paz et al. 2005 [30], Gomes et al. 2004 [31], Claesson et al. 2017 [12], Rolph et al. 2001 [32], Sundqvist et al. 1998 [33], Vigil et al. 1997 [34], Sjogren et al. 1997 [35], Gomes et al. 1996 [36], Debelian et al. 1995 [37], Fukushima et al. 1990 [38], Qi et al. 2016 [39], Fernandes et al. 2014 [40], Tennert et al. 2014 [41], Chugal et al. 2011 [42], Ledezma-Rasillo et al. 2010 [43], Zhang et al. 2010 [22], Mindere et al. 2010 [44], Cogulu et al. 2008 [45], Chu et al. 2005 [46], Chavez de Paz et al. 2004 [47], Siqueira et al. 2004 [48], Hirshberg et al. 2003 [21], Tang et al. 2003 [49], Xia et al. 2003 [50], Pinheiro et al. 2003 [51], Siqueira et al. 2002 [52], Peters et al. 2002 [53], Sunde et al. 2002 [20], Siqueira et al. 2002 [54], Ercan et al. 2006 [55], Molander et al. 1998 [56], Ruviere et al. 2008 [57], Sundqvist et al. 1992 [58], Brauner and Conrads 1995 [59], Assed et al. 1996 [60], Hancock et al. 2001 [61], Esteves et al. 2017 [19], Persoon et al. 2017 [18];
- seven articles for the tertiary outcome: Ercan et al. 2006 [55], Chugal et al. 2011 [42], Tennert et al. 2014 [41], Fernandes et al. 2014 [40], Rolph et al. 2001 [32], Gomes et al. 2004 [31], Lysakowska et al. 2016 [25].

K agreement between the two screening reviewers was 0.625 (Table 2). The K agreement was based on the formulas of the *Cochrane Handbook for Systematic Reviews* [62].

**Figure 1:**
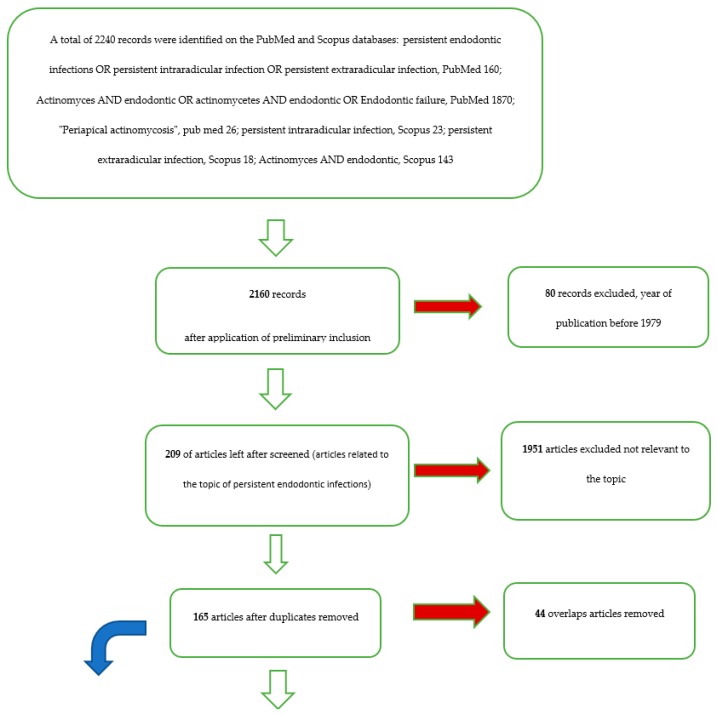

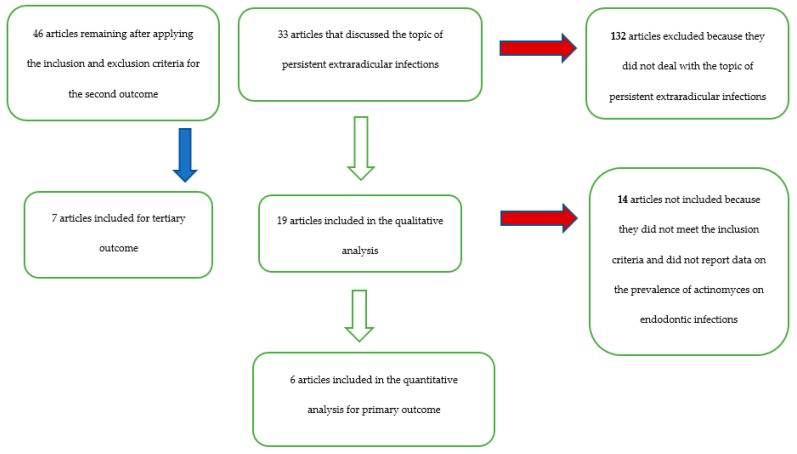
Flow chart of the different phases of the systematic review.

The entire selection and screening procedures are described in the flow chart (Figure 1).

### 3.1. Study Characteristics and Data Extraction

The extracted data included the magazine (author, data, and journal), the bacterium species of the genus *Actinomyces* investigated (genus, species, and number of dental elements with the presence of the bacterium), the number of samples examined, type of sample (necrotic or vital tooth, endodontic canal, tooth in pulpitis or apical periodontitis, tooth previously treated endodontically, tooth with failure subject to extraction or endodontic surgery), the number of samples per pathology with the presence of *Actinomyces*, and the method used for bacterium identification (PCR or culture).

If data on the prevalence in single studies were reported only for the individual species of *Actinomyces* and the overall data were not present or it was not possible to obtain them, the data pertaining to the species were considered for the purpose of the meta-analysis, which in the single study presented the higher prevalence. If the data were reported as a percentage, the number was calculated through the use of proportions.

The data extracted for the tree outcomes are shown in Table 3 and Table 4.

### 3.2. Risk of Bias

The risk of bias was assessed through the Newcastle–Ottawa case-control scale, modified for the cumulative meta-analysis. The results are reported in detail in Table 5. For each category, a value of one to three was assigned (one = low and three = high).

Studies presenting a high risk of bias were not included in the meta-analyses. Articles with high Bias risk were excluded from the scale and eliminated during the inclusion phase. Other articles were excluded because they presented the same data and samples for the outcomes investigated. The risk of bias assessment for the 46 articles included was conducted by the first reviewer (M.D.).

**Figure 2:**
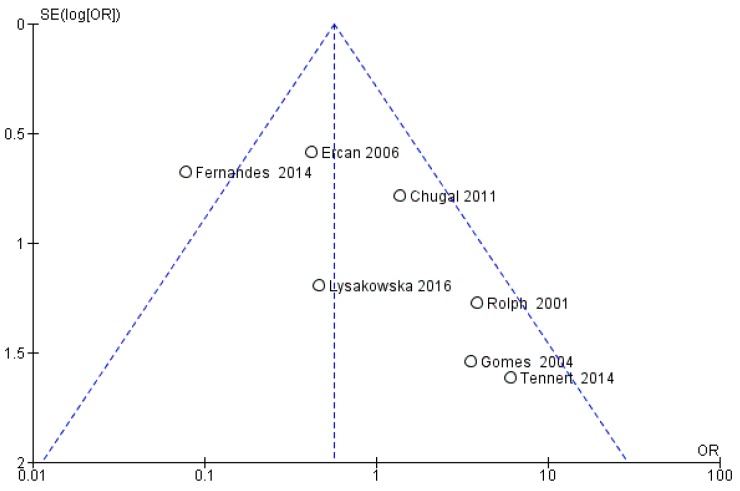
Funnel plot of the evaluation of heterogeneity of tertiary outcomes.

The risk of bias between the studies is considered very high for the primary and secondary outcome; in fact, the heterogeneity that emerges from the meta-analysis shows an *I*^2^ equal to 88.09% for the primary outcome and 90.96% for the secondary outcome. For the tertiary outcome, the risk of bias between the studies was assessed through the funnel plot for the seven articles included (Figure 2).

### 3.3. Meta-Analysis

**Figure 3:**
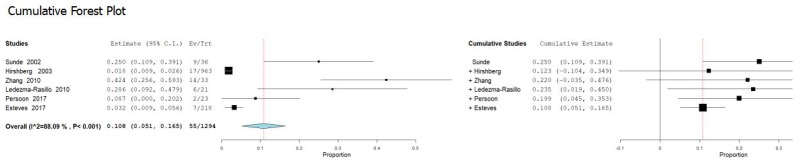
Forest plot of the random effects model of the cumulative meta-analysis of the primary outcome.

The heterogeneity of the primary outcome (number of teeth with the presence of a persistent extraradicular infection in which the presence of *Actinomyces* has been ascertained) was high with an *I*^2^ equal to 88.09%. For this reason, a random effects model was used. The cumulative meta-analysis presents an overall pooled proportion (*I*^2^ = 88.09%, *p* value < 0.001) of 0.108 (0.051, 0.165) with a ratio between events and samples examined equal to 55/1294 (Figure 3).

**Figure 4:**
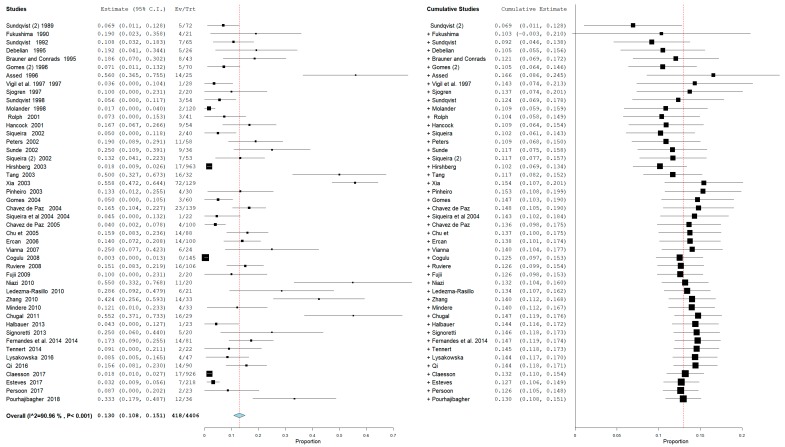
Forest plot of the random effects model of the cumulative meta-analysis of the secondary outcome.

For the secondary outcome, the heterogeneity was very high with an *I*^2^ equal to 90.96%. For that reason, a random effects model was used. The cumulative meta-analysis presents a pooled proportion (*I*^2^ = 90.96%, *p* value < 0.001) of 0.130 (0.108, 0.151) with a ratio between events and samples examined equal to 418/4406 (Figure 4).

**Figure 5:**
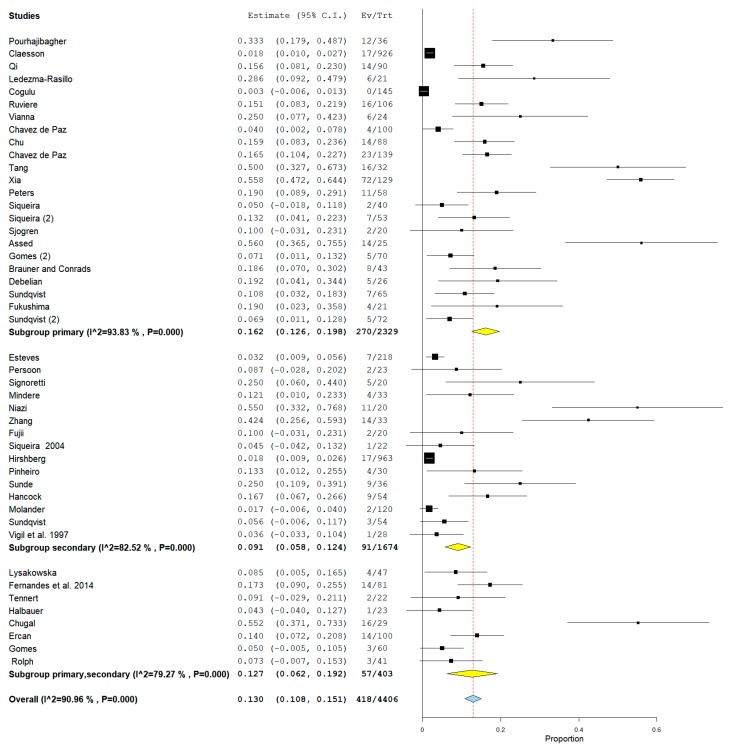
Forest plot of the random effects model of the meta-analysis of the secondary outcome (subgroups primary, secondary, and primary/secondary).

In consideration of the high heterogeneity of the studies, an analysis of the subgroups for the secondary outcome was also conducted. Studies were divided into primary, secondary, and primary/secondary, based on whether they investigated the presence of bacteria of the genus *Actinomyces* in teeth with primary or secondary infection or in both. The results are reported in Figure 5.

**Figure 6:**
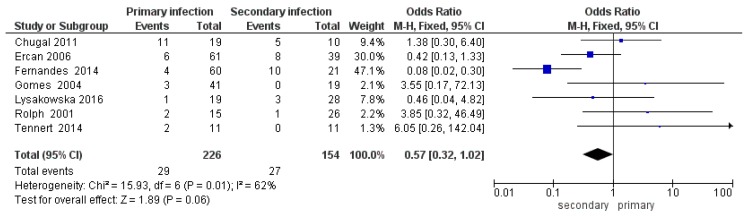
Forest plot of the fixed effects model of the meta-analysis of the tertiary outcome.

For the tertiary outcome—difference in the prevalence of bacteria of the genus *Actinomyces* between primary endodontic infections and secondary endodontic infections—the comparison showed average heterogeneity among the studies, with an *I*^2^ equal to 62%. Results reported in Figure 6 show that the rate of *Actinomyces* infection was higher in secondary than in primary endodontic infection (OR = 0.57, 95%CI: (0.32, 1.02)).

## 4. Discussion

Bacteria of the genus *Actinomyces* (optional anaerobic Gram-positive, rods) are very often identified in both primary and secondary endodontic infections. In addition, the literature places such microorganisms among the main causes of persistent extra-root infections together with the bacteria of the genus *Propionibacterium*. Both genera belong to the order of *Actinomycetes* and can colonize the external root surface, subsequently giving persistence of the lesion independently of the endodontic infection [4].

The path of penetration of *Actinomyces* within the root canal system is not entirely clear. *Actinomyces* is a normal commensal of the oral bacterial flora, and its penetration inside the endodontum may depend on the loss of the coronal seal. The most important cases of actinomycosis are associated with histories of complicated root canal treatments, but *Actinomyces* can, however, also be found in periapical lesions in which a root canal treatment has never been performed.

*Actinomyces* destroy local tissue and replace it with inflammatory and abscess tissue. The granules are generally yellowish in color but can be white-green or green-brown and are formed by masses of filamentous microorganisms that extend in a radiant way and sometimes appear calcified [64].

The purpose of these aggregations is to resist the action of the immune system, the microorganisms in these formations are resistant to phagocytosis creating a microenvironment favorable to their growth and acting as a barrier to the action of antibiotics [65].

Teeth subjected to a radiographically correct endodontic retreatment [66], but with the presence of radiographic radiolucent lesion, fistula with drainage of purulent material, are suspected of persistent extraradicular infection that can evolve into a cervical facial form, characterized clinically by skin lesions with hardened area with multiple abscesses containing grainy tissue, which tend to form cavities and drain onto the skin with purulent material containing granules described as “sulfurous” (the name sulfurous derives from the yellowish coloring of the yellow bacterial filamentous aggregates) [67].

There is also the possibility that the extraradicular infection may affect the maxillary sinuses, and that the infection may continue even after the extraction of the dental element if the patient is immune deficient, giving a picture of sinusitis [68].

In the last years, some reviews of the literature focusing on the microbiological aspects of endodontic infections have been performed. A narrative review performed by Yoo et al. in 2019 identified four types of biofilm (intracanal, extraradicular, periapical, and biomaterial-centered biofilms), indicating *Actinomices* and *Propionibacterium* as the main culprits of the extraradicular biofilms, and they can also colonize filling materials [69].

In 2016, Sakko et al. investigated the presence of bacteria in various endodontic lesions analyzing the associations most found in the literature and the probable path of penetration, suggesting possible ways of treatment [70].

Foaud, in 2019, focused on the microbiological aspects of teeth subjected to traumatic injuries, identifying *Actinomyces* among the bacteria mainly involved and also focusing on the use of antibiotics in the case of traumatized teeth [71].

Prada et al. reported in 2019 how most authors identified *E. faecalis* as the main microorganism associated with endodontic failures, noting, however, that recent studies isolate, to a greater extent, other bacteria such as *Fusobacterium nucleatum* and *Propionibacterium* [15].

Previously, Zhang et al. included in 2015 10 studies on *E. faecalis*, analyzing a total of 927 teeth and concluded that such bacteria are more highly correlated with persistent intraradicular infections compared with untreated chronic periapical periodontitis [72]. Very recently, Manoli et al. published a systematic review including 26 studies that used new sequencing technologies aiming at redesigning a new map of the bacterial taxa associated with endodontic infections. Such review identified bacteria with a higher prevalence in infections but found no significant difference in the three groups analyzed (primary apical periodontitis, secondary apical periodontitis, and apical abscess) [73]. Such literature reviews, conducted over the past five years, have only marginally investigated the role of *Actinomyces* in persistent extraradicular infections. Only few of them focused on extraradicular bacterial biofilms, and only a few of these report data on the prevalence of *Actinomyces* in endodontic infections. This review differs from the previous ones because it focuses on the prevalence of bacteria of the genus *Actinomyces* in endodontic infections.

Results of the present review showed that bacteria of the genus *Actinomyces* most frequently found in endodontic infections are: *A. naeslundii*, *A. israelii*, *A. viscosus*, A. *odontolyticus*, *A meyeri*, *A. gerencseriae*, *A. radicidentis*, and *A. halioti*; the latter three bacteria have been identified and researched with a much lower frequency (Table 3). In addition, the prevalence of bacteria of the genus *Actinomyces* in the teeth with endodontic failures subject to surgical treatment is 55 out of 1294 teeth examined with a ratio of 0.108 (Figure 2). In some studies, the presence of *Actinomyces* on teeth with endodontic failures refractory to non-surgical therapy reached a ratio of 14 out of 33 teeth in a study by Zhang et al. 2010 [22] and 6 out of 36 in a study by Sunde et al. 2002 [20].

Estevens, on the other hand, reported slightly different data, on a total of 218 peri-apical lesions. The presence of bacterial colonies was identified only in 64 biopsies and only 7 showed the presence of actinomycosis. Furthermore, women resulted to be the most affected gender, while the site most affected was the jaw. All cases had peri-apical lesions persisting to root canal treatment and were therefore subject to surgical therapy (extraction or apicectomy) [19]. Hirshberg identified 17 typical colonies of actinomycosis out of 963 apical biopsies with a higher incidence in males and maxilla [21].

The second half of the analysis reported a prevalence of *Actinomyces* in all the included studies of 418 presences on 4406 teeth with an overall ratio of 0.130. Given the high heterogeneity of the studies, subgroups were also investigated. In the subgroups of studies investigating both primary and secondary infections, heterogeneity decreased with *I*^2^ = 79.27% and an overall prevalence of 14.1% with a ratio of 57/403. The data are in partial agreement with the cumulative meta-analysis for the secondary outcome.

The meta-analysis for the tertiary outcome relates to the prevalence of *Actinomyces* between primary and secondary infections in studies that investigated both conditions. The forest plot reports statistically significant data with fewer events (presence of *Actinomyces*) in teeth prone to primary infections. All studies intersect the non-effect line except for Fernandes et al. 2014 [40]. Studies in favor of a lower presence of *Actinomyces* bacteria in primary infections were Ercan et al. 2006 [55], Lysakowska et al. 2016 [25], and Fernandes et al. 2014 [40].

## 5. Conclusions

In conclusion, we can affirm that the bacteria of the genus *Actinomyces* are present in both primary and secondary intraradicular infections with a prevalence (ratio between teeth with *Actinomyces* and teeth with infection) ranging from 0.091 to 0.130, depending on the subgroups analyzed. Furthermore, they are responsible for many of the cases of extra-root infections with persistence of the lesions even following correct endodontic reprocessing.

In cases of persistence of intraradicular and extraradicular infections, many authors agree on establishing that the only solution is surgical, with operations of apicectomy or extraction of the dental element, to avoid complications such as facial cervical actinomycosis and facial imperfections.

## Figures and Tables

**Table 1:** Complete overview of the search methodology. Records identified by databases: 2240.

Database-Provider	Keywords	Search Details	Number of Records	Number of Records) after Restriction by Year of Publication (Last 40 Years)	Number of Remaining Articles Related to the Topic of Bacteria in Endodontic Infections	Articles after Removing Overlapping Articles	Number of Articles Remaining after Applying the Inclusion and Exclusion Criteria for the Secondary Outcome	Number of Articles Included for Tertiary Outcome (Difference in the Prevalence of Bacteria of the Genus *Actinomices* Between Primary Endodontic Infections and Secondary Endodontic Infections)	Number of Remaining Articles Pertaining to the Topic of Persistent Extraradicular Infections	Number of Articles Focusing on the Role of Actinomycetes a on Extraradicular Persistent Lesions	Number of Articles Included for the Primary Outcome
**Pub-med**	persistent endodontic infections OR persistent intraradicular infection OR persistent extraradicular infection	(persistent (All Fields) AND endodontic (All Fields) AND (“infection” (MeSH Terms) OR “infection” (All Fields) OR “infections” (All Fields))) OR (persistent (All Fields) AND intraradicular (All Fields) AND (infection” (MeSH Terms) OR “infection” (All Fields))) OR (persistent (All Fields) AND extraradicular (All Fields) AND (“infection” (MeSH Terms) OR “infection” (All Fields)))	160	158	42						
**Pub-med**	*Actinomyces* AND endodontic OR *actinomycetes* AND endodontic OR Endodontic failure	(“*actinomyces*” (MeSH Terms) OR “*actinomyces*” (All Fields)) AND endodontic (All Fields) OR (“actinobacteria” (MeSH Terms) OR “actinobacteria” (All Fields) OR “actinomycetes” (All Fields)) AND endodontic (All Fields) OR (Endodontic (All Fields) AND failure (All Fields))	1870	1814	111						
**Pub-med**	“Periapical actinomycosis”	“Periapical actinomycosis” (All Fields)	26	11							
**Scopus**	persistent intraradicular infection	TITLE-ABS-KEY (persistent AND intraradicular AND infection)	23	23	14						
**Scopus**	persistent extraradicular infection	TITLE-ABS-KEY (persistent AND extravascular AND infection)	18	18	15						
**Scopus**	*Actinomyces* AND endodontic	TITLE-ABS-KEY (actinomyces AND endodontic)	143	136	27						
**Total records**			2240	2160	209	165	46	7	33	19	6

**Table 2:** K agreement calculation, Po = 0.848 (Proportion of agreement), Pe = 0.595 (Agreement expected), K agreement = 0.625 (<0 no agreement, 0.0–0.20 slight agreement, 0.21–0.40 fair agreement, 0.41–0.60 moderate agreement, 0.61–0.80 substantial agreement, 0.81–1.00 almost perfect agreement). The K agreement was calculated from the 33 articles to include six articles with the application of the inclusion and exclusion criteria for primary outcome.

		Reviewer 2	Reviewer 2	Reviewer 2	
		Include	Exclude	Unsure	Total
**Reviewer 1**	Include	6	0	0	6
**Reviewer 1**	Exclude	3	22	2	27
**Reviewer 1**	Unsure	0	0	0	0
	Total	9	22	2	33

**Table 3:** The data on the prevalence of the various bacteria of the genus *Actinomyces* in the various studies included for the three outcomes are reported.

Author, Date, Journal	Actinomyces	Type of Tooth or Root, of Dental Treatment, or Endodontic Pathology				Number of Teeth or Channels or Periapical Tissue in Which the Presence of Actinomycetes Has Been Identified		Total Number of Teeth or Channels or Periapical Tissue in Which the Presence of Actinomycetes Was Investigated	Identification Method of Bacterial Species
[24] Pourhajibagher et al. 2018 *Photodiagnosis and photodynamic therapy*	*A. naeslundii*	12	root canal samples	12/36		12		36	culture
[25] Lysakowska et al. 2016 *International endodontic journal*	*A. naeslundii*	0/19	primary endodontic infections	1/19		4		47	culture
		2/28							
	*A. meyeri*	1/19	secondary treatment	3/28					
		1/28							
[26] Halbauer et al. 2013 *Coll Antropol*	*A. meyeri*	1/23	chronical apical periodontitis (*n* = 17 untreated teeth)	17		1		23	culture
			chronical apical periodontitis (*n* = 6 retreatments)	6					
[23] Signoretti et al. 2013 *Journal of endodontics*	*A. naeslundii*	2/13	persistent apical lesions associated with well-performed endodontic retreatment (*n* = 13 cyst *n* = 7 granuloma)	20		5		20	culture
		3/7							
	*A. meyeri*	1/13							
		1/7							
[27] Niazi et al. 2010 *Journal of endodontics*	*A. gerencseriae*	1/20	20 refractory endodontic lesions (5/9 with abscesses and 6/11 without abscesses)	20		11		20	PCR
	*A. massiliensis*	1/20							
	*A. meyeri*	1/20							
	*A. radicidentis*	1/20							
	*A. israelii*	1/20							
	*Actinomyces sp.*	7/20							
[28] Fujii et al. 2009 *Oral microbiology and immunology*	*A. israelii*	2/16	infection lesions with apical periodontitis 20 (16 without sinus tract, 5 with sinus tract)	2/20		2		20	PCR
		0/5							
[29] Vianna et al. 2007 *Oral microbiology and immunology*	*A. naeslundii*	6/24	human necrotic root canals	6/24		6		24	PCR
[30] Chavez de Paz et al. 2005 *Oral surgery, oral medicine, oral pathology, oral radiology, and endodontics*	*A. israelii*	1/100	teeth with apical periodontitis	4/100		4		100	PCR
	*A. meyerii*	2/100							
	*A. naeslundii*	2/100							
	*A. odontolyticus*	4/100							
	*Actinomyces spp*	1/100							
[31] Gomes et al. 2004 *Oral microbiology and immunology*	*Actinomyces meyerii*	3/0	41 primary infection	3/60		3		60	PCR
		0/19	19 endodontic failure						
[12] Claesson et al. 2017 *Anaerobe*	*A. radicidentis*	16/926	root canal samples			17		926	PCR
	*A. haliotis*	1/926							
[32] Rolph et al. 2001 *Journal of clinical microbiology*	*A. naeslundii*	2/15	2/15 primary endodontic infections			3		41	culture
		0/26							
	*A. viscosus*	1/15							
		0/26	1/26 refractory cases of endodontic infections						
	*A. israelii*	0/15							
		1/26							
[33] Sundqvist et al. 1998 *Oral surgery, oral medicine, oral pathology, oral radiology, and endodontics*	*A. israelii*	3/54	54 teeth with failed endodontic treatment			3		54	culture
[34] Vigil et al. 1997 *Journal of endodontics*	*A. odontolyticus*	1/28	28 refractory endodontic cases requiring surgical intervention			1		28	culture
[35] Sjogren et al. 1997 *International endodontic journal*	*A. naeslundii*	1/20	20 apical periodontitis			2		20	culture
	*A. odontolyticus*	1/20							
	*A. israelii*	2/20							
[36] Gomes et al. 1996 *J dental*	*A. naeslundii*	2/70	necrotic pulp			5		70	culture
	*A. viscosus*	3/70							
	*A. israelii*	4/70							
	*A. meyeri*	5/70							
[37] Debelian et al. 1995 *Endodontics & dental traumatology*	*A. israelii*	5/26	26 teeth with asymptomatic apical periodontitis			5		26	culture
	*A. meyeri*	1/26							
	*A. naeslundii*	2/26							
	*A. odontolyticus*	1/26							
[38] Fukushima et al. 1990 *Journal of endodontics*	*A. israelii*	2/21	21 untreated cases			4		21	culture
	*A. viscosus*	2/21							
	*A. meyeri*	1/21							
	*A. naeslundii*	1/21							
[39] Qi et al. 2016 *Int Endod J*	*A. naeslundii*	14/90	primary endodontic infections			14		90	PCR
	*A. israelii*	2/90							
	*A. viscosus*	0/90							
[40] Fernandes et al. 2014 *Microb Pathog*	*Actinomyces spp*	irreversible pulpitis (0–27) pulp necrotic teeth with apical periodontitis (4–33)				4/60	14	81	PCR
		apical periodontitis associated with a root-filled tooth (10–21)				10/21			
Tennert et al. 2014 [41]	*A. viscosus*	1/11	primary infection			2/11	2	22	PCR
		0/11							
	*A. naeslundii*	1/11	secondary/persistent infection			0/11			
		0/11							
[42] Chugal et al. 2011 *J Endod*	*Actinomyces* spp.	11/19	primary endodontic infections			11/19	16	29	PCR
		5/10	secondary infections			5/10			
[43] Ledezma-Rasillo et al. 2010 *J Clin Pediatr Dent*	*A. israelii*	4/21	primary teeth with necrotic pulps			6		21	culture
	*A. naeslundii*	2/21							
[22] Zhang et al. 2010 *Chin J Dent Res*	*A. israelii (21%)*	persistent apical periodontitis				14		33	PCR
	*A. viscosus (42%)*								
[44] Mindere et al. 2010 *Stomatologija*	*A. odontolyticus*	1/33	root-filled teeth with asymptomatic persisting periapical lesions			4		33	culture
	*A. israelii*	1/33							
	*A. viscosus*	2/33							
[45] Cogulu et al. 2008 *Oral Surg Oral Med Oral Pathol Oral Radiol Endod*	*A. israelii*	acute apical periodontitis (deciduous 20, permanent 22)				0		145	PCR
		chronic apical periodontitis (deciduous 35, permanent 28)							
		exacerbated apical periodontitis (deciduous 24, permanent 16)							
[46] Chu et al. 2005 *J Endod*	*A. israelii (7%) (14%)*	3/45	primary endodontic infections with exposed 45; primary endodontic infections with unexposed 43			14		88	culture
		6/43							
	*A. meyeri (13%) (19%)*	6/45							
		8/43							
	*A. odontolyticus (11%) (19%)*	5/45							
		8/43							
[47] Chavez de Paz et al. 2004 *Int Endod J*	*A. israelli*	2/23	apical periodontitis			23		139	PCR
	*A. meyeri*	7/23							
	*A. naeslundii*	3/23							
	*A. odontolyticus*	6/23							
	*A. radicidentis*	0/23							
	*A. viscosus*	0/23							
	*Actinomyces* spp.	1/23							
[48] Siqueira et al. 2004 *Oral Surg Oral Med Oral Pathol Oral Radiol Endod*	*A. israelii*	0/22	root-filled teeth with persistent periradicular lesions			1		22	PCR
	*A. radicidentis*	1/22							
[21] Hirshberg et al. 2003 *Oral Surg Oral Med Oral Pathol Oral Radiol Endod*	*Actinomyces spp*	persistent periapical lesions				17		963	histology
[49] Tang et al. 2003 *J Dent*	*odontolyticus (31.3%)*	10/32	primary root canal infections			16		32	PCR
	*A. meyeri (9.4%)*	3/32							
	*A. naeslundii (9.4%)*	3/32							
	*A. israelii (6.3%)*	2/32							
	*A. gerencseriae (3.1%)*	1/32							
[50] Xia et al. 2003 *J Endod*	*A. israelii (23.7%)*	31/129	primary root canal infections (41/51)			72		129	PCR
	*A. naeslundii (8.5%)*	11/129	abscesses (22/48)						
	*A. viscosus (32.1%)*	41/129	cellulitis (9/31)						
[51] Pinheiro et al. 2003 *Oral Microbiol Immunol*	*A. naeslundii*	2/30	teeth with endodontic failure			4		30	PCR
	*A. odontolyticus*	1/30							
	*A. viscosus*	1/30							
[52] Siqueira et al. 2002 *Int Endod J*	*A. israelii*	root canal infections, necrotic pulps				2		40	PCR
[53] Peters et al. 2002 *Int Endod J*	*Actinomyces* spp.	3/58	primary endodontic infections			11		58	culture
	*A. odontolyticus*	11/58							
	*A. meyeri*	6/58							
[20] Sunde et al. 2002 *J Endod*	*A. israelii*	6/36	periapical lesions refractory to endodontic therapy			9		36	culture
	*A. meyeri*	3/36							
	*A. viscosus*	7/36							
	*Actinomyces species*	1/36							
	*A. naeslundii*	5/36							
[54] Siqueira et al. 2002 *J Endod*	*A. gerencseriae*	4/53	primary root; canal infections			7		53	PCR
	*A. israelli*	2/53							
	*A. naeslundii*	0/53							
	*A. odontolyticus*	1/53							
[55] Ercan et al. 2006 *Biotechnol. & Biotechnol. Eq.*	*A. odontolyticus*	4/61	61 had necrotic pulp tissues (primary infection)		6/61	14		100	culture
		4/39							
	*A. meyeri*	2/61							
		0/39	39 had a history failed endodontic treatment (secondary infection)		8/39				
	*A. naeslundii*	0/61							
		4/39							
[56] Molander et al. 1998 *Int Endod J*	*Actinomyces* spp.	100 root-filled teeth with radiographically verified apical periodontitis (*n* = 2)				2		120	culture
		20 root-filled teeth without signs of apical periodontitis							
[57] Ruviere et al. 2008 *J Dent Child (Chic)*	*A. viscosus*	0/55	55 root canals of primary teeth with irreversible pulpitis			16		106	PCR
		0/51							
	*A. naeslundii genospecies 1*	0/55							
		2/51							
	*A. odontolyticus*	3/55							
		10/51	51 root canals of primary teeth with necrotic pulp and apical periodontitis						
	*A. israelii*	0/55							
		10/51							
	*A. gerencseriae*	2/55							
		10/51							
[58] Sundqvist et al. 1992 *Oral Microbiol Immunol*	*Actinomyces* sp., *‘1*	1/65	nonvital teeth with periapical lesions			7		65	culture
	*A. israelii*	7/65							
	*A. meyeri*	1/65							
	*A. naeslundii*	3/65							
	*A. odonlotyticus*	1/65							
	*A. viscosus*	1/65							
[59] Brauner and Conrads 1995 *Int Endod J*	*Actiuomyccs* spp.	5/19	19 root canal (*n* = 6)			8		43	culture PCR
		2/24							
	*A. israelii*	1/19	24 periapical granuloma (*n* = 2)						
		0/24							
[60] Assed et al. 1996 *Endod Dent Traumatol*	*A. viscosiis*	chronic apical periodontitis				14		25	immunofluorescence
[61] Hancock et al. 2001 *Oral Surg Oral Med Oral Pathol Oral Radiol Endod*	*Actinomyces* spp.	chronic apical periodontitis in teeth with endodontic failure				9		54	culture
[19] Esteves et al. 2017 *Braz Dent J*	*Actinomyces*	persistent periapical lesions (cysts, granulomas or abscess)				7		218	histology
[18] Persoon et al. 2017 *Clin Oral Investig*	*Actinomyces*	apical periodontitis and refrained from endodontic treatment				2		23	PCR
[63] Sundqvist et al. 1989 *J Endod*	*A. israelii*	1/72	necrotic pulps and apical periodontitis			5		72	culture
	*A. meyerii*	2/72							
	*A. viscosus*	1/72							
	*A. odontolyticus*	1/72							

**Table 4:** Tertiary outcome (difference in the prevalence of bacteria of the genus *Actinomyces* between primary endodontic infections and secondary endodontic infections).

Author, Date, Journal	Species	Primary Endodontic Infections		Secondary/Persistent Infection	
		event	total	event	total
[42] Chugal et al. 2011 *J Endod*	*Actinomyces* spp.	11	19	5	10
	tot	11	19	5	10
[55] Ercan et al. 2006 *Biotechnol. & Biotechnol. Eq.*	*A. odontolyticus*	4	61	4	39
	*A. naeslundii*	0	61	4	39
	*A. meyeri*	2	61	0	39
	tot	6	61	8	39
[41] Tennert et al. 2014 *J Endod*	*A. viscosus*	1	11	0	11
	*A. naeslundii*	1	11	0	11
	tot	2	11	0	11
[40] Fernandes et al. 2014 *Microb Pathog*	*Actinomyces* spp.	4	60	10	21
	tot	4	60	10	21
[32] Rolph et al. 2001 *Journal of clinical microbiology*	*A. naeslundii*	2	15	0	26
	*A. israelii*	0	15	1	26
	*A. viscosus*	1	15	0	26
	tot	2	15	1	26
[31] Gomes et al. 2004 *Oral microbiology and immunology*	*Actinomyces meyerii*	3	41	0	19
	tot	3	41	0	19
[25] Lysakowska et al. 2016 *International endodontic journal*	*A. naeslundii*	0	19	2	28
	*A. meyeri*	1	19	1	28
	tot	1	19	3	28

**Table 5:** Assessment of risk of bias within the studies (Newcastle–Ottawa scale) with scores 7 to 12 = low quality, 13 to 20 = intermediate quality, and 21 to 24 = high quality.

		Selection			Comparability		Exposure		Score
Reference	Definition of Cases	Representativeness of Cases	Selection of Controls	Definition of Controls	Comparability of Cases and Controls on the Basis of the Design or Analysis	Ascertainment of Exposure	Same Method of Ascertainment for Cases and Controls	Non-Response Rate	
[24] Pourhajibagher et al. 2018 *Photodiagnosis and photodynamic therapy*	3	3	0	0	0	3	3	0	12
[12] Claesson et al. 2017 *Anaerobe*	2	3	0	0	0	3	3	0	11
[19] Esteves et al. 2017 Braz *Dent J*	3	3	0	0	3	0	0	0	9
[18] Persoon et al. 2017 *Clin Oral Investig*	3	3	0	0	1	3	1	0	11
[25] Lysakowska et al. 2016 *International endodontic journal*	3	3	3	3	2	2	3	0	19
[39] Qi et al. 2016 *Int Endod J*	3	3	0	0	0	3	3	0	12
[40] Fernandes et al. 2014 *Microb Pathog*	3	3	2	3	2	3	3	0	19
[41] Tennert et al. 2014 *Journal of endodontics*	2	3	3	2	2	3	3	0	18
[26] Halbauer et al. 2013 *Coll Antropol*	1	3	3	1	2	3	3	0	16
[23] Signoretti et al. 2013 *Journal of endodontics*	2	1	2	2	2	3	3	0	15
[42] Chugal et al. 2011 *Journal of endodontics*	2	2	1	2	2	3	3	0	15
[22] Zhang et al. 2010 *Chin J Dent Res*	2	2	2	2	2	2	2	0	14
[43] Ledezma-Rasillo et al. 2010 *The Journal of clinical pediatric dentistry*	3	1	2	2	2	2	3	0	15
[44] Mindere et al. 2010 *Stomatologija*	2	2	0	0	0	2	2	0	8
[45] Cogulu et al. 2008 *Oral Surg Oral Med Oral Pathol Oral Radiol Endod*	3	3	0	0	0	3	3	0	12
[27] Niazi et al. 2010 *Journal of endodontics*	3	1	3	3	2	1	3	0	16
[28] Fujii et al. 2009 *Oral microbiology and immunology*	2	2	0	0	0	2	2	0	8
[57] Ruviere et al. 2008 *J Dent Child (Chic)*	3	3	2	2	2	3	3	0	18
[29] Vianna et al. 2007 *Oral microbiology and immunology*	3	2	3	2	2	2	3	0	17
[55] Ercan et al. 2006 *Biotechnol. & Biotechnol. Eq*.	3	2	3	3	3	3	3	0	20
[46] Chu et al. 2005 *Journal of endodontics*	3	2	3	3	3	2	3	0	19
[30] Chavez de Paz et al. 2005 *Oral surgery, oral medicine, oral pathology, oral radiology, and endodontics*	3	3	3	3	2	3	3	0	20
[31] Gomes et al. 2004 *Oral microbiology and immunology*	2	2	2	3	3	3	3	0	21
[47] Chavez de Paz et al. 2004 *International endodontic journal*	3	3	3	3	3	2	3	0	20
[48] Siqueira et al. 2004 *Oral surgery, oral medicine, oral pathology, oral radiology, and endodontics*	3	1	0	0	0	3	2	0	9
[21] Hirshberg et al. 2003 *Oral Surg Oral Med Oral Pathol Oral Radiol Endod*	3	2	0	0	0	2	2	0	9
[49] Tang et al. 2003 *J Dent*	3	3	0	0	0	3	1	0	10
[50] Xia et al. 2003 *J Endod*	3	3	0	0	0	3	0	0	9
[51] Pinheiro et al. 2003 *Oral Microbiol Immunol*	3	3	0	0	0	3	0	0	9
[52] Siqueira et al. 2002 *Int Endod J*	2	3	0	0	0	3	0	0	8
[53] Peters et al. 2002 *Int Endod J*	2	3	0	0	0	3	0	0	8
[20] Sunde et al. 2002 *Journal of endodontics*	2	2	2	2	3	2	3	0	16
[54] Siqueira et al. 2002 *J Endod*	3	3	1	0	0	0	3	0	10
[61] Hancock et al. 2001 *Oral Surg Oral Med Oral Pathol Oral Radiol Endod*	3	3	2	0	0	0	3	0	11
[32] Rolph et al. 2001 *Journal of clinical microbiology*	3	3	3	3	3	2	3	0	20
[33] Sundqvist et al. 1998 *Oral surgery, oral medicine, oral pathology, oral radiology, and endodontics*	2	2	2	2	2	2	3	0	15
[56] Molander et al. 1998 *International endodontic journal*	3	3	0	0	0	2	3	0	11
[34] Vigil et al. 1997 *Journal of endodontics*	3	2	0	0	0	3	2	0	10
[35] Sjogren et al. 1997 *International endodontic journal*	2	2	2	2	3	2	3	0	16
[36] Gomes et al. 1996 *J dental*	3	2	0	0	0	2	2	0	9
[60] Assed et al. 1996 *Endod Dent Traumatol*	2	3	0	0	0	3	0	0	8
[59] Brauner et al. 1995 *International endodontic journal*	3	2	0	0	0	2	2	0	9
[37] Debelian et al. 1995 *Endodontics & dental traumatology*	2	2	2	2	3	2	2	0	15
[58] Sundqvist et al.1992 *Oral microbiology and immunology*	2	2	2	0	0	0	2	0	8
[38] Fukushima et al. 1990 *Journal of endodontics*	2	1	2	0	0	0	2	0	7
[63] Sundqvist et al. 1989 *Journal of endodontics*	3	3	3	0	0	0	2	0	11
